# Learning with Patient Campaigners About a German Drug Scandal**

**DOI:** 10.1002/bewi.70014

**Published:** 2026-02-27

**Authors:** Jesse Olszynko‐Gryn, Anja Suter, Edmund Bolger, Birgit Nemec

**Affiliations:** ^1^ Max‐Planck‐Institut für Wissenschaftsgeschichte Boltzmannstraße 22 14195 Berlin Germany; ^2^ Institut für Geschichte der Medizin und Ethik in der Medizin Charité ‐ Universitätsmedizin Berlin Thielallee 71 14195 Berlin Germany; ^3^ Independent Filmmaker, Berlin Germany; ^4^ Fakultätszentrum für transdisziplinäre historisch‐kulturwissenschaftliche Studien Universität Wien Kolingasse 14‐16 1090 Wien Austria

**Keywords:** disability, Duogynon, ethics, health activism, inclusion, oral history

## Abstract

The West German drug Duogynon was internationally marketed as a “hormone pregnancy test” (HPT) between the 1950s and 1980s. In the late 1960s it came under suspicion for inducing miscarriage, spina bifida, and a spectrum of birth defects similar to those caused by the sedative thalidomide. In contrast to thalidomide, medical consensus did not form around the teratogenicity of Duogynon and many people who identify as Duogynon‐affected continue to campaign for recognition. The informal use of Duogynon as an abortion pill adds a further layer of shame, secrecy, and silence. In this article, we reflect on the value of oral history and the ethics of inclusion within a larger research project that investigates the rise and fall of HPTs, globally. We ask what collaborating with patient campaigners in a more participatory mode than is typical of archival research can contribute to the historical understanding of the Duogynon affair and other drug scandals.

## 1 The Duogynon Affair

Today, it can be hard to believe that doctors ever prescribed pills as a pregnancy test. But from the 1950s to the 1980s, millions of women worldwide were prescribed diagnostic drugs that ruled out gestation by inducing menstruation‐like bleeding; the absence of blood confirmed pregnancy. Hormone pregnancy tests (HPTs), of which Duogynon was the first and most commercially successful, were an innovation of the West Berlin pharmaceutical company Schering AG (subsequently acquired by Bayer) in 1950. Schering positioned Duogynon (marketed as Primodos in Britain and later rebranded as Cumorit) as a cheaper and more convenient alternative to the expensive and cumbersome urine test, which involved injecting laboratory animals, usually a frog, with a woman's urine. As with the ulcer drug misoprostol (Cytotec), Duogynon was widely used, off label, as an abortion pill.^1^

In the late 1960s, Duogynon and other HPTs came under suspicion for inducing miscarriage, spina bifida, and a spectrum of birth defects akin to those caused by the sedative thalidomide (marketed in West Germany as Contergan).^2^ Amidst growing controversy, Schering voluntarily withdrew Duogynon from the British and West German markets in 1978 and 1981, respectively (by then HPTs had already been banned or otherwise restricted across the Global North). Meanwhile, the parents of children with congenital disabilities whose mothers had taken an HPT while pregnant organized to take legal actions against Schering (and the UK government), calling Duogynon a “second thalidomide”.^3^

These actions came to naught and there the story might have ended. In the early 2010s, however, the examination of previously inaccessible archival records breathed new life into the long‐dormant media and legal campaigns and provided the impetus for new biomedical and historical research, and the reanalysis of old epidemiological data.^4^ The net effect has been to reopen the still unresolved debate over the teratogenicity (ability to cause birth defects) of Duogynon, with the most recent review asserting that HPTs are teratogenic by same mechanism as misoprostol in cases of failed abortion (blood vessel damage with lack of oxygen during fetal development).^5^ Though the individual risk is estimated to have been low, HPTs were so popular that thousands of children in Britain and West Germany would have been affected.^6^

Despite this renewed attention, the history of HPTs remains obscure and poorly understood. This is especially true when it comes to the lived experience of the people who self‐identify as HPT‐affected. This is because most historical analysis, including our own, has tended to emphasize the power and agency of “governments, medical communities, and pharmaceutical industries” to the neglect of “impacted families”.^7^ A case in point is historian Niklas Lenhard‐Schramm's book, *Die Duogynon‐Affäre* (2024), which expands on a report he produced for the German Ministry of Health (BMG) in 2022.^8^ The book follows a *drug trajectories* approach that reconstructs the medical, legal, and regulatory histories in great detail but has little to say about the families.^9^ Nor were they involved in the research process, a fact that is described as a “serious shortcoming” in a recent critical review of the book.^10^

This article takes inspiration from recent projects in Belgium, Canada, and the UK that draw on oral history and disability history to center the knowledge, expertise, and agency of people who have been affected by thalidomide and diethylstilbestrol (DES), a transplacental carcinogen.^11^ It takes to heart the “nothing about us without us” dictum of disability studies as well as an important recent call on health historians to “do less harm”.^12^ More concretely, it reflects on the value of oral history and the ethics of inclusion within a larger project that studied the rise and fall of HPTs, globally.^13^

## 2 The “Risky Hormones” Project

The “Risky Hormones” project is an ongoing collaboration between Jesse Olszynko‐Gryn and Birgit Nemec in partnership with two patient advocacy groups: the Association for Children Damaged by Hormone Pregnancy Tests (ACDHPT) and Netzwerk Duogynon e.V.^14^ It grew out of and built on a collaboration between Jesse Olszynko‐Gryn and Marie Lyon, Chair of the ACDHPT, which dates back to 2015, when Olszynko‐Gryn's doctoral thesis on the history of pregnancy testing was requested as evidence by a governmental review of HPTs.^15^ In 2017, Olszynko‐Gryn organized a conference in Cambridge, where Birgit Nemec presented her preliminary research on West Germany. Following her presentation, Birgit Nemec was contacted by members of Netzwerk Duogynon and their supports, to develop a research collaboration in Germany.

Both groups generously offered to share their knowledge and expertise. In consultation with them, Birgit Nemec and Jesse Olszynko‐Gryn conceptualized a binational research project, which from 2020 to 2025 received funding from the UK Arts and Humanities Research Council (AHRC) and the German Research Foundation (DFG) through the then newly established UK‐German Funding Initiative in the Humanities.^16^ In this period, the Co‐PIs relocated from Glasgow and Heidelberg, where they were initially based, to Berlin. Meanwhile, the UK government commissioned a second, independent review (the “Cumberlege Review”) and the German Ministry of Health (BMG) commissioned the report by Lenhard‐Schramm that would form the basis for his aforementioned book.^17^

The Cumberlege Review, which reported in 2020, examined HPTs alongside sodium valproate, a teratogenic epilepsy drug, and vaginal mesh. It collected oral testimonies from ACDHPT members, concluded the system had “failed” them, and called for an apology.^18^ In contrast, the BMG excluded members of Netzwerk Duogynon from the review process and the report, which exonerated the Federal Health Office (BGA), a predecessor organization of the BMG, sparked criticism from network members.^19^ These developments added another layer of urgency to the need for collaboration on the German side of the project, which intensified as a result. It is on this aspect of the project that the rest of the article focuses.

## 3 Netzwerk Duogynon

Netzwerk Duogynon represents around 660 people (including 130 full members) who identify as Duogynon‐affected and are united in their campaign for recognition, redress, and compensation. Many need care and several have had children who died young. Duogynon was not marketed in East Germany so, with few exceptions, most grew up in the West. The demographic distribution of the group is bimodal, with the parents now past retirement age and their children passing through midlife. As with most such groups, the majority of members are comparatively passive, often due to health limitation, but nevertheless stay connected via the newsletter and chat group. A smaller, more active subgroup takes care of meetings, lobbying, legal support, and media relations.

Network members, many of whom have struggled financially and socially throughout their lives, seek closure and justice. The statute of limitations is a major obstacle to financial compensation but an apology and an independent review that involved the campaigners (as with Cumberlege) would be meaningful.^20^ Meanwhile, patient campaigners lack formal recognition as people who have been harmed by Duogynon. The official position has always been that the birth defects would have happened anyway and it is often impossible to prove causation in individual cases.^21^ Crucially, this marks Duogynon‐affected people apart from many (though by no means all) thalidomide and DES survivors, for whom the cause of their normally incredibly rare conditions (phocomelia and adenocarcinoma, respectively) is not in question.^22^

Our point of contact with the network was André Sommer, a primary school teacher from Pfronten, a small Bavarian town on the edge of the Allgäu Alps, and the group's spokesperson. The first person we interviewed, Sommer is also the protagonist of a short (unpublished) essay film we made together that explores his engagement with and, in some cases, exploitation by the media. A Duogynon‐child, he was born in 1976 with bladder exstrophy, a rare condition that required numerous surgeries. Sommer vouched for us and facilitated our broader engagement with network members. Through him, we put out a call for interview participants.

The response was enthusiastic, with many apparently wanting to tell their stories and go on record. In light of higher‐than‐expected demand, we made a decision to give all group members the opportunity to opt into participation. This significantly increased the number of interviews from eight, as initially planned, to a total of twenty‐nine, almost a quarter of all full members. These were conducted in 2023 and 2024 by Birgit Nemec and Anja Suter, a research associate on the project, who traveled around Germany, in some cases also with Eddie Bolger, the project's filmmaker, to visit group members in their homes. Self‐selection favored interview partners who were eager to narrate their lives, in some cases for the first time beyond their community or to anyone.

## 4 The Value of Oral History

Oral history has long been valued as a useful tool for historians of health, medicine, and disability.^23^ For one, it facilitates the integration of perspectives that are often absent, marginalized, or misrepresented in archival records, including those of patients and disabled people. Beyond generating new lines of evidence for research purposes, oral history is also a tool for establishing trust and building relationships. The outcome we aspired in the “Risky Hormones” project to was mutual learning about the past, or “making history together.”^24^

In this regard, the project follows in the traditions of community oral history, public history, and community archival projects, among others.^25^ Recent, proximate examples include the (UK) Heritage Lottery Fund‐supported “Thalidomide Stories” project, which innovatively provided training for survivors to interview one another, and the Manchester‐based “NHS at 70” project, which collected stories from patients, staff, and members of the public to create a digital archive of the UK's National Health Service.^26^ In the collaborative, participatory spirit of such projects, though on a smaller scale, we set out to collaborate with members of Netzwerk Duogynon in a meaningfully inclusive, empathetic, and accountable mode.^27^

Because the German side of the project was based at the Charité, a medical school, it was required to go through a rigorous, two‐stage ethics review, which included the development of a comprehensive, four‐page consent form and resulted in the adoption of a trauma‐informed approach (to minimize the risk of retraumatization and other forms of distress).^28^ At a later stage, we participated in a full‐day tailored training course provided by the (UK) Oral History Society, and we initiated a partnership with Oral‐History.Digital, a research platform that hosts audiovisual interview collections.^29^ Many network members expressed a desire to share their life stories with a wider public. So, in consultation with them, we linked up with Lamm & Kirch, a small independent design studio, to develop a more accessible, public‐facing website based on the interviews.

Likewise in consultation with Sommer and other network members, we adopted a flexible approach to interviewing, data storage, anonymization, and so on; one that we hoped would be able to accommodate a range of preferences. We also transferred as much control as possible over the medium (video or audio), location (in situ or in Berlin), anonymization or pseudonymization, and level of access (from completely open to highly restricted). Beyond the requirements of the Charité ethics review and the consent form, interview partners also had the right to opt out at any time, ask us to remove their interviews from the repository, as well as to review and make edits. A typical interview lasted between one and three hours and usually took place at the home of the network member, often but not only due to limited mobility related to disability or age. A few interview partners, however, traveled to Berlin to give their testimonies at the Charité. Some had previously granted interviews to journalists but most had not.

Of course, we brought our own research agenda and questions to the interview. We wanted to know when and under what circumstances the mothers first encountered Duogynon, when they first learned about the possible implications for pregnancy and the fetus, and how they first became involved in campaigning. But, as practitioners well know, “interviews rarely go as planned” and ours were no exception.^30^ Network members directed the conversation, including by asserting the tone and pace, and by deciding which stories to tell, and how they wanted to tell them. There were often stretches of up to twenty minutes that hardly needed or even allowed for intervention; our job was to listen carefully and sympathetically, and to keep recording.

Holding a nonjudgmental space for Duogynon‐affected people to recollect highly personal aspects of their medical encounters or private lives in their own time, without interruption or correction, emerged as an especially crucial feature of the project.^31^ We did not question or challenge the political views of the campaigners, the legitimacy of their grievances, or (in many cases) their understandable skepticism toward medical expertise and health governance at large.^32^

The vistas opened up by the interviews, we suggest, invite new ways of understanding the Duogynon affair. For one thing, the interviews bring into focus categories that are largely absent from previous historical accounts (including our own) but highly significant for the affected people themselves. These include “solidarity,” “silence,” “darkest life episodes,” “fight,” “suicide,” “guilt,” “loss of trust,” “loneliness,” “self‐help,” and “gratitude”. When it comes to identity, the lexicon of network members spans “disability,” “malformation,” “birth defect,” “impairment,” and “handicap”. Listening to them taught us about the variable terminology they use to describe themselves—typically without reference to academic disability studies or theory—and why they preferred one term over another, or none at all.^33^

As newly cocreated primary sources, the interviews were of immediate use to the “Risky Hormones” project. In this sense, they resemble the *research interview* as thematized in this special issue. On the other hand, we also conceived them as life story interviews intended for archival deposition. This repository, now hosted by Oral‐History.Digital, contains a wealth of information that we hope will be of more general relevance beyond the specific interests of the project. It is, in effect, a collection of *disability stories* that speak to major themes in the social history and disability history of West Germany, including parenthood, childhood, family life, education, employment, sexuality, health care, and activism.^34^ We further hope the group members will recognize themselves in the repository and, more especially, in the public‐facing website.

The repository is intended to supplement not replace archival sources. These remain indispensable to anyone interested in the Duogynon affair but are severely limited when it comes to the patient's perspective. Crucially, our interviews facilitate “triangulation across sources,” as Dmitriy Myelnikov puts it in this issue.^35^ Oral history better equips researchers to cross‐examine archival records that are evasive about shameful topics such as abortion or informal activities such as the off‐label use of drugs. In other words, interviews, when they yield frank speech, can help historians interpret archival silences and know when to read between the lines. Interviews may be constructed, but so too are archival records, and oral history can be just as illuminating and is not more problematic than archival research, which, though never innocent or neutral, is still often construed as the “historian's gold standard”.^36^

## 5 The Informal Archive

The single largest archival collection available to Duogynon researchers, including campaigners, is a vast store of documents held by the Berlin state archive (*Landesarchiv*), the bulk of which were generated by an unsuccessful legal action brought against Schering in the late 1970s. A second, much smaller, but invaluable (though currently inaccessible) collection is held by the Schering archive, also in Berlin. Other relevant collections, which include correspondence with German government officials, can be found in London, at the National Archives and in the Wellcome Library. Auction websites function to some extent as commercial archives for ephemera, including promotional material for HPTs.

**Figure 1:**
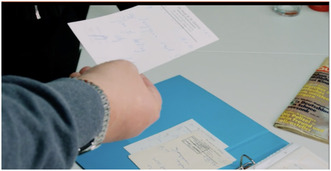
A patient campaigner shows us a folder with a magazine, medical notes, and other documentation in the course of an interview. Photo: Edmund Bolger.

Finally, there exists a highly dispersed privately held collection of newspaper and magazine clippings, family snapshots, medical records, prescriptions, legal reports, correspondence with members of parliament, drug packaging, and so on. As Myelnikov notes, again in this issue, “going through the documents during the conversation can be highly evocative and useful, both in bringing back memories and in highlighting the disconnect between what one remembers and what the documents say” (Figure 1).^37^ This was our experience too.

Many Duogynon‐parents, as we learned from their children, still kept the documents relating to their medical experiences and campaigning, often in a well‐organized folder or box in the basement or attic. Sommer, for example, informed us that his father presented him with one such folder in 2009. For some 30 years, it had languished in the family's attic. It contained newspaper clippings, medical reports, photographs, correspondence, and, crucially, a list of telephone numbers for campaigners that had been compiled by Sommer's mother in the 1970s. Sommer's parents had never or rarely discussed with him the suspected cause of his condition or his mother's campaigning, so the folder came as a revelation. A few months after being presented with the folder, Sommer used the list to reconnect with his mother's associates, and their now grown‐up children, with some of whom he prepared and new lawsuit and, in 2019, reactivated the campaign under the banner of Netzwerk Duogynon.

Our encounter with Sommer further disclosed a pill box gifted to him by a Duogynon‐mother whose daughter had been born with only one kidney and severe malformations of the urethra. We later interviewed the mother, who preferred to remain anonymous, and she recounted the story of the object. At the time of her daughter's birth, she was living in Switzerland. There she befriended another woman whose child was born just a few days later in the same hospital with similar malformations. She eventually moved away but the two women remained in contact. Seventeen years later, during a social visit, the visiting mother produced the pill box from her handbag and confessed that she was not able to rid herself of the suspicion that the cause of her daughter's malformations was an HPT that her gynecologist had prescribed to her in Switzerland: “That's when it dawned on me: I took that too! I recognize the pill box […] I was prescribed exactly the same thing.”^38^

**Figure 2:**
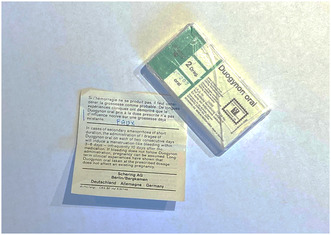
Plastic pill box and package insert with handwritten annotation. Courtesy of André Sommer. Photo: Birgit Nemec.

The visiting mother claimed to have carried the pill box in her handbag until that moment. The object itself dates from 1973, and is notable for still carrying the wording on the packaging that Duogynon, if taken as prescribed, would “not affect an existing pregnancy”. The visiting mother had annotated the insert in blue ink: “FAUX” (WRONG); this minimalist commentary boldly stakes a clear epistemic claim in a debate that is unresolved to this day (Figure 2). It was also this moment which prompted our interview partner to research the history of Duogynon, eventually leading her to join the network. The visiting mother gifted her the pill box, which she in turn gifted to Sommer.

## 6 Ignorance, Silence, and Family Secrets

Ignorance about Duogynon, a topic we have written about elsewhere, also emerged as a recurring theme across the interviews.^39^ It structured recollections of pregnancy, maternity, childhood, family relations, and campaigning. For each of the campaigners we talked to, personal, public, and medical ignorance about the drug and its effects contributed to what they continue to experience as lack of closure. Many disclosed, often for the first time to anyone, a heavy silence regarding the cause of their condition that persisted for decades between themselves, on the one hand, and relatives, carers, and doctors, on the other.^40^

Many women did not know what they were taking when they ingested Duogynon tablets. Ursula Krämer from Nuremberg, whose daughter was born in 1978 with a perceptual disorder, recalled that she “wasn't thinking about pregnancy at the time and the doctor just said, yes, to be on the safe side, she gave me the two tablets. I should take them, if no bleeding comes, I can assume I am pregnant, and otherwise everything would be normal.”^41^ Thea Hermann from East Berlin was puzzled that her mother was given the tablets by her family doctor as a test, whereas her aunt, who had advised her mother on reproductive decisions, had just a year earlier taken the same product explicitly to terminate an early pregnancy.^42^

It took Hermann, as with many Duogynon‐children who subsequently joined the campaign, a long time and much detective work to learn about the debate over Duogynon and its effects. Based on conversations she overheard as a child between her mother and grandparents, Hermann later began to assume that no one in her family openly spoke about her impairment, a malformation of the left eye, because they associated the “tainted” test with an attempted abortion. The absence of knowledge, information, and public debate over Duogynon as well as the shame, embarrassment, and stigma of abortion seems to have contributed to an aura of silence within families.

Sommer's parents hid not only their knowledge of Duogynon from him for a long time, but also the fact of their own involvement in campaigning on his behalf. Hermann's mother kept silent also because she had arranged and administered a drug that was not licensed in East Germany; she had obtained Duogynon from her sister, who, as a famous musician, was permitted to cross the Iron Curtain.^43^ From Beate Helmling of Mannheim, we gained insights into the dynamics of a particular mother‐daughter relationship that was affected at least as much by secrecy, shame, and distrust than by any disability or health condition, in her case (as with Sommer), bladder exstrophy: “I was always told that I was a whim of nature. Well, my mum was not honest with me, she always kept it from me. Everyone, I know now in retrospect, everyone knew that she took the pills, except me.”^44^

Helmling, who became dependent on care following multiple difficult surgeries on her lower abdomen, did not suspect Duogynon until a Stern TV programme about the drug featured Sommer and she connected the dots: “I was sitting on the couch with my daughter one evening, and then she said, “Mom, look, there's bladder exstrophy, your disease”, and of course I put my cell phone down and watched with excitement.” Following this revelation, the first thing she did, “of course”, was call her mother: “She was already 79 or 80 at the time, I cannot remember. And then I asked her about this abortion tablet and she did not want to talk about it at first. She kept it to herself for years because I was probably far too strong for her to tell me the truth. Well, my mom wanted to abort me, that was the case back then. […] And of course I was devastated because I had been lied to and deceived all my life.”^45^

Helmling's mother apparently took the tablets as an abortifacient. Already the mother of two children and in an unstable relationship with an international student from Iran, she hoped to terminate the pregnancy but did not tell anyone about her use of Duogynon.^46^ After her daughter was born with “impairments”, as Helmling refers to them, the secrecy only deepened. We can only speculate about the effects on her mother of “ke[eping] it to herself” for all those years. Confessing to her daughter later in life came “as a relief” to Helmling's mother, but was devastating for Helmling, whose self‐understanding collapsed in that moment.^47^

## 7 The Ethics of Inclusion

To date, Niklas Lenhard‐Schramm's book, *Die Duogynon‐Affäre* (2024), which grew out of his report for the BMG (2022), is the most thorough history of HPTs. Weighing in at over 400 pages, it makes unprecedented use of the state archives to reconstruct the medical, legal, and regulatory history. As with the report, the book exonerates the Federal Health Office (BGA), the BMG's predecessor organization, for failing to ban the controversial drug, due to legal uncertainties, when it might have done so.

And yet, by cleaving so closely to the state regulatory history and excluding patient campaigners from his analysis, Lenhard‐Schramm misses a crucial part of the story. Indeed, it is because of them that the affair has not faded into obscurity. That both our projects received state funding is down to the persistence of two generations of patient campaigners, including Sommer and his UK counterpart, Marie Lyon. These are people with agency who, like their predecessors in the late 1970s, have made history.

As historian Marc von Miquel puts it in his critical review of *Die Duogynon‐Affäre*, Lenhard‐Schramm's investigation largely omits the engagement of civil society, namely, the “injured party and their advocacy group,” and, with it, the emergence in 1978 of a “community of Duogynon critics” and their allies in the media. This, von Miquel continues, is a serious omission because the emergence of a pharma‐critical public in the 1960s marked a “turning point in society's approach to medicine.” He further identifies the decision not to include Netzwerk Duogynon in the research process as a “serious shortcoming”. It would have been “appropriate,” he concludes, for the research to have been guided by the “ethical principles of the right to participation and transparency,” not least because participatory research can yield “significant gains in knowledge.”^48^

As Sommer recently put it in an email to Birgit Nemec, Duogynon‐affected people “want to be involved [in the research]. They want to tell their life stories. They want to be heard.”^49^ It is to the ethical principles of inclusion, responsibility, and accountability, that we have attempted however imperfectly to adhere to in the “Risky Hormones” project. We hope the effort will yield significant gains in knowledge and, moreover, that these will contribute to a more rounded historical understanding of the Duogynon affair—one that reaches beyond the governmental, medical, legal, and corporate actors, whose perspectives are so well preserved in state archives, to more fully reckon with the agency of the people whose lives have been most directly and profoundly affected by Duogynon.

## Conflict of Interest

The authors declare no conflict of interest.

## Interviews

Birgit Nemec, Anja Suter, Edmund Bolger, and André Sommer, Pfronten, 10 March 2023

Birgit Nemec and Ursula Krämer, Nuremberg, 4 November 2023

Birgit Nemec and Beate Helmling, Mannheim, 5 January 2024

Birgit Nemec and Thea Hermann, Berlin, 5 March 2024

Anja Suter and Anon., Munich, 15 March 2024

**Access:** https://portal.oral‐history.digital/rh‐oh/de

## Data Availability

The data that support the findings of this study are available from the corresponding author upon reasonable request.

## Footnotes

1
Olszynko‐Gryn et al., 2018; Nemec and Olszynko‐Gryn 2022; Olszynko‐Gryn 2023a, 145–168.

2
Gal et al. 1967.

3
Nemec and Olszynko‐Gryn 2022, 80.

4
Vargesson 2016; Commission on Human Medicines 2017; Brown et al., 2018; Lenhard‐Schramm 2018, 2022, 2024; Olszynko‐Gryn et al., 2018; Heneghan et al., 2019; Cumberlege 2020; Heneghan and Aronson 2021; Danielsson et al. 2023; Nemec and Olszynko‐Gryn 2022; Tümmler et al. 2014; Wildblood 2024; Danielsson and Ritchie 2025.

5
Danielsson and Ritchie 2025.

6
Danielsson and Ritchie 2025, 17.

7
Van Kerckhove and Claes 2024, 537.

8
Lenhard‐Schramm 2022, 2024.

9
Lenhard‐Schramm 2024. On the approach: Gaudillière 2005.

10
Von Miquel 2025.

11
Chisholm 2019; Blue 2021; Van Kerckhove and Claes 2024. On disability as a useful analytic category for historians of medicine and science: Linker 2013; Kudlick 2018; Hogan 2022; Mills et al. 2024.

12
Charlton 2000; Mills et al. 2024; Thompson and Smith 2025.

13 The best overview of ethics in oral history is Sheftel and Zembrzycki 2016. See also Larson 2013; Gidharry 2021; Chansky 2024.

14 See the websites of ACDHPT, https://primodos.org, and Netzwerk Duogynon, https://duogynonopfer.de.

15
Commission on Human Medicines 2017.

16 On power relations and the ethics of project funding in oral history: Gidharry 2021.

17
Cumberlege 2020; Lenhard‐Schramm 2022.

18
Mahase 2020; Lewens 2024.

19 See von Miquel 2025.

20
Jurkschat 2025.

21
Lewens 2024.

22 On the problem of recognition in the absence of medical documentation: Parle and Wimmelbücker 2020; Olszynko‐Gryn 2023b, Olszynko‐Gryn et al. 2023; Wimmelbücker and Kar 2023.

23 See Thompson 1991; Tomes 1991; Hirsch 1995; Bornat et al., 2000; Fisher 2011; Pelka 2012.

24
Snow and Whitecross 2022.

25 See, for example, Smith 2010; Popple et al. 2020; Gidharry 2021.

26 See “Thalidomide Stories: An Oral History of Thalidomide Survivors”, https://thalidomidesociety.org/oral‐histories/; Snow and Whitecross 2022.

27 See Thompson and Smith 2025.

28 On trauma and oral history: Cramer 2020; Cave and Sloan 2014; Gidharry 2021; Chansky 2024.

29 See Pagenstecher 2024.

30
Sheftel and Zembrzycki 2013, 4.

31 On the importance of holding space for interview partners: Fern et al. 2025.

32 On medical skepticism and distrust in healthcare, which can often be well founded: Conis 2013; Jones and Reverby 2022.

33 See Watson 2002; Mills and Bouk 2024.

34 On disability in West German history: Bösl 2009; Stoll 2017; Rössel 2022. For a recent assessment of the state of the art and future directions: Kömpe et al. 2025.

35
Myelnikov 2026.

36
Myelnikov 2026. For critical approaches to archival research: Schwartz and Cook 2002; Jimmerson 2009; Stoler 2010; Caswell et al. 2017.

37
Myelnikov 2026.

38 Anja Suter and Anon., interview, Munich, 15 March 2024, https://portal.oral-history.digital/rh-oh/de/interviews/rh-oh0017; all translations by the authors.

39
Nemec and Olszynko‐Gryn 2022.

40 On intrafamilial silence in the case of DES: Van Kerckhove and Claes 2024.

41 Birgit Nemec and Ursula Krämer, Nuremberg, 4 November 2023, https://portal.oral-history.digital/rh-oh/de/interviews/rh-oh0014.

42 Birgit Nemec and Thea Hermann, Berlin, 5 March 2024, interview, https://portal.oral‐history.digital/rh‐oh/de/interviews/rh‐oh0008.

43 On border crossers in the GDR see Niederhut 2005; on artistic internationalism in East Germany see Hutchinson 2024.

44 Birgit Nemec and Beate Helmling, interview, Mannheim, 5 January 2024, https://portal.oral‐history.digital/rh‐oh/de/interviews/rh‐oh0007.

45 Nemec and Helmling, interview. On the importance of television in the case of DES: Van Kerckhove and Claes 2024, 554.

46 On Iranian students in West Germany: Slobodian 2012.

47 Nemec and Helmling, interview. On the historical significance of family secrets: Cohen 2013; Nelson 2022; Van Kerckhove and Claes 2024.

48
Von Miquel 2025.

49 André Sommer to Birgit Nemec, email 27 August 2025.
